# Health Outcomes and Lifestyle in a Sample of People With Multiple Sclerosis (HOLISM): Longitudinal and Validation Cohorts

**DOI:** 10.3389/fneur.2018.01074

**Published:** 2018-12-12

**Authors:** Tracey J. Weiland, Alysha M. De Livera, Chelsea R. Brown, George A. Jelinek, Zoe Aitken, Steve L. Simpson, Sandra L. Neate, Keryn L. Taylor, Emily O'Kearney, William Bevens, Claudia H. Marck

**Affiliations:** ^1^Neuroepidemiology Unit, Centre for Epidemiology and Biostatistics, Melbourne School of Population and Global Health, The University of Melbourne, Melbourne, VIC, Australia; ^2^Biostatistics Unit, Centre for Epidemiology and Biostatistics, Melbourne School of Population and Global Health, The University of Melbourne, Melbourne, VIC, Australia; ^3^Menzies Institute for Medical Research, University of Tasmania, Hobart, TAS, Australia; ^4^Disability and Health Unit, Centre for Health Equity, Melbourne School of Population and Global Health, The University of Melbourne, Melbourne, VIC, Australia

**Keywords:** cohort study, multiple sclerosis, lifestyle, disability, risk factor

## Abstract

**Objective:** To report the methodology and summary data of the Health Outcomes and Lifestyle In a Sample of people with Multiple sclerosis (HOLISM) longitudinal and validation cohorts. We report (1) data on participation, socio-demographics, disease characteristics, medication use, modifiable lifestyle risk factor exposures, and health outcomes of the HOLISM longitudinal cohort 2.5-years post enrolment; (2) attrition at this 2.5-year wave; and (3) baseline characteristics of the associated HOLISM validation cohort.

**Methods:** The HOLISM longitudinal study recruited people internationally with self-reported diagnosed multiple sclerosis (MS) through web 2.0 platforms and MS society newsletters. Participants, first recruited in 2012, were invited 2.5-years later to participate in a follow-up survey. At both time points, participants completed a comprehensive online questionnaire of socio-demographics, modifiable lifestyle exposures, and health outcomes using validated and researcher-designed tools. The same methodology was used to recruit a new sample: the HOLISM validation cohort. Characteristics were explored using summary measures.

**Results:** Of 2,466 people with MS at baseline, 1,401 (56.8%) provided data at 2.5-year follow-up. Attrition was high, likely due to limited amount of contact information collected at baseline. Completion of the 2.5-year wave was associated with healthier lifestyle, and better health outcomes. Participants completing follow-up had diverse geographical location, were predominantly female, married, unemployed or retired. At 2.5-year follow-up, nearly 40% were overweight or obese, most were physically active, non-smokers, consumed little alcohol, used vitamin D/omega-3 supplements, and 42% reported current disease-modifying drug use. Thirty percentage of reported cane or gait disability, while 13% relied on major mobility supports (Patient Determined Disease Steps). Approximately half the respondents reported a comorbidity, 63% screened positive for clinically significant fatigue (Fatigue Severity Scale), and 22% screened positive for depression (Patient Health Questionnaire-9). The validation cohort's characteristics were mostly consistent with previously reported HOLISM baseline data.

**Conclusions:** Exploring prospective associations of modifiable environmental/behavioral risk factors with health outcomes in this international longitudinal sample of people with MS will be beneficial to MS research. Impacts of attrition and selection bias will require consideration. The validation cohort provides opportunity for replication of previous findings, and also for temporal validation of predictive models derived from the HOLISM cohort.

## Background

Multiple sclerosis (MS) is the most common debilitating neurological disorder affecting young adults (1), impacting at least 2.3 million people globally in 2013 (2). MS is characterized by a variety of symptoms including impaired muscle power, mobility, vision, and symptoms of pain, fatigue, depression, cognitive dysfunction, loss of bladder control, and sexual dysfunction. Recently, a wealth of research has identified behavioral and environmental modifiable lifestyle factors that may attenuate disease severity, symptoms and/or comorbidities. The prospective relationships between these modifiable exposures and outcomes, and the inter-relationships between variables, however, are yet to be fully elucidated. This underscores the importance of longitudinal panel studies of health outcomes and associated factors.

While the etiology of MS progression remains uncertain, growing evidence suggests an important role for behavioral and environmental factors including the modifiable lifestyle risk factors smoking and low vitamin D levels (3, 4). MS progression may involve inflammatory, metabolic and neurodegenerative disease processes (5–8) which could be moderated by changes in lifestyle. Although randomized controlled trials (RCTs) assessing the impact of lifestyle modification on overall disease course are lacking (9), there is evidence from observational studies that healthy lifestyle is associated with fewer comorbidities (10), as well as less MS symptoms, and slower disability accumulation and disease progression (11–13), suggesting that secondary prevention of deterioration may be feasible. The collection of comprehensive, longitudinal lifestyle, and environmental exposure data for people with MS is imperative to identify potential secondary preventive approaches for this population.

We previously explored associations between modifiable lifestyle risk factors and health outcomes in people with MS in the baseline cohort of the study, Health Outcomes and Lifestyle In a Sample of People with Multiple Sclerosis (HOLISM) (14). In a suite of publications, our baseline findings have suggested that there are clinically meaningful, cross-sectional associations between modifiable lifestyle risk factors and health outcomes in people with MS (11, 15–19). At 2.5 years after the initial baseline recruitment, we invited participants to complete a follow-up survey to examine how modifiable lifestyle risk factors, clinical disease features, sociodemographic characteristics, and medication use are prospectively associated with health outcomes for people with MS

This paper reports in detail the methodology of the 2.5-year follow-up, and provides an overview of participation, attrition, and cohort characteristics. Additionally, it documents the baseline characteristics of a validation sample collected using the same methodology, contemporaneously to the HOLISM longitudinal sample 2.5-year wave.

## Methodology

### Participants and Data Collection

Adults self-reporting a doctor diagnosis of MS were recruited for HOLISM baseline between May and August 2012 (14). We have previously reported only on those participants indicating a formal diagnosis of MS by a medical doctor; however, people with a possible MS diagnosis were also permitted to complete the survey. Participants were recruited over 15 weeks using websites, mailing lists of MS societies, and web 2.0 platforms such as blogs, forums, and Facebook pages created specifically for people with MS, as well as other social media such as Twitter. Participants provided consent and completed the survey online hosted by SurveyMonkey.

In November 2014, 2.5 years after the baseline survey, we invited all participants (including those who indicated a diagnosis of clinically isolated syndrome or possible MS at baseline) to complete a follow-up survey. An email inviting participation contained a link to the survey webpage presenting them with a participant information sheet, which they were asked to read before providing consent. Indicating consent was necessary to continue participation in the survey. The survey was open between 16 Nov 2014 and 30 Sept 2015, with 75% of the sample completing the survey within the first month. In order to capture those participants with contact details that were no longer current, and to recruit new participants into a validation cohort (20), we also recruited participants via websites, a mailing list, and web 2.0 platforms such as blogs, forums, Facebook pages and groups, and Twitter accounts created specifically for people with MS. Baseline data were matched to follow-up surveys using participant IDs, and other identifying information including name, date of birth and location, and those participants who had not participated in the baseline HOLISM survey were identified. Their data will be analyzed separately as the validation cohort.

The purpose of recruiting this validation cohort was two-fold: (1) For replicability of our original findings from the HOLISM cohort; and (2) For temporal validation (21) of predictive models derived from the HOLISM cohort. Temporal validation, involves the application of derived and internally validated models to a sample similar to the derivation sample but recruited subsequently (20–22).

Ethics approval was granted by St Vincent's Hospital Melbourne Human Research Ethics Committee (LRR055/12) and subsequently by The University of Melbourne's Health Sciences Human Ethics Sub-Committee (HESC 1545102).

### Data Collection Tools

The survey comprised 100 items at baseline and 111 items at 2.5-year follow-up; however, skip logic enabled participants to skip items not relevant to them. Survey completion took approximately 40 min, participants were able to take breaks and even continue the survey on another day if needed.

To allow longitudinal analysis, we predominantly used the same tools employed at baseline. As described previously (14), HOLISM surveys included, where possible, questionnaires that were psychometrically sound and had been tested in comparable populations. If validated questions or tools were unavailable, measures were developed by the research team. Further validated tools not used at baseline were added at the 2.5-year follow-up, including the Patient Health Questionnaire-9 (23), the Pearlin Mastery Scale (24), the Performance Scale-Vision Component (PS-V) (25, 26), the Economic Ladder Question (27), and health service utilization questions (28). Additional researcher-designed tools included at 2.5-year follow-up measured sun exposure, oily fish consumption, and the influence of MS on employment.

The survey consisted of the following domains:

#### Contact Details

Two email addresses and an email address for a next of kin were requested. These items were mandatory before being able to progress to the next part of the survey.

#### Socio-Demographics

Data were collected for date of birth, sex, current city, and country of residence (to derive latitude), country of birth, cultural background, marital status, number of biological, adopted, and step children, employment status, education level, height, and weight. Additionally, we included a researcher-derived categorical question to explore influence of MS on employment, with nine response options available: “MS has not impacted on my employment; I have had to cut back my hours by ~25% due to MS; I have had to cut back my hours by approximately 50% due to MS; I have had to cut back my hours by approximately 75% due to MS; I have had to retire completely due to MS; I have not progressed in my career due to MS; I have had to quit or change jobs due to MS; I have had to change my day to day tasks at work due to MS; I have to take increased sick leave due to MS.” For the purpose of this paper these categories were collapsed to form a binary variable: MS impacted on my employment: yes/no.

Cultural background was categorized using the Australian Standard Classification of Cultural and Ethnic Groups (29), with an additional free-text “other” response available. Body mass index (weight/height^2^) was calculated and classified using the WHO classification system (30). Data for socioeconomic status were captured using the Economic Ladder Question: “If you compare yourself to others in your country, and imagine the poorest people on the first step and the richest people on the ninth step, where would you place yourself today?.” While this is traditionally administered as a pictorial vertical ladder (27), here it was administered as a horizontal 9-point visual analog scale with descriptors, “poorest,” “average,” and “richest” from left to right.

#### Diagnostic History

Participants' diagnostic history included confirmation of MS diagnosis by a medical doctor, year of diagnosis, year of first symptoms, type of MS upon diagnosis and at time of survey completion, number of relapses observed by the participant in the preceding 12 and 60 months, and number of doctor-diagnosed relapses in the preceding 12 and 60 months. Participants were provided with a definition of a relapse based on that given in the North American Research Committee on Multiple Sclerosis (NARCOMS) survey. Some items for diagnostic history were modified from the NARCOMS enrolment questionnaire (obtained from NARCOMS study authors).

#### Level of Disability

The Patient-Determined Disease Steps (PDDS) was used to assess level of disability. This is a self-reported analog of the Expanded Disability Status Scale (EDSS) which is commonly used by neurologists to assess gait disability (31). The PDDS is scored ordinally from 0 (normal) to 8 (bed bound) with detailed descriptors and definitions. For analyses we collapsed these into three groups: “normal/mild” (scores 0–2 indicating no walking impairment); “moderate” (scores 3–5 indicating gait disability or single cane); and “severe” (scores 6–8 indicating requirement for two canes, a wheelchair or bed).It correlates strongly with the EDSS and Functional System score (32) and is considered a practical tool to assess changes in disability over time (33). The patient-derived Multiple Sclerosis Severity Score (P-MSSS) (34) was used as an additional indicator of disease status. Essentially analogous to the MSSS (35), it estimates the disease duration-adjusted level of disability as measured by PDDS, such that the same PDDS after a shorter disease duration will realize a higher P-MSSS than the same PDDS later in disease. Higher scores on the P-MSSS denote greater disease severity. In calculating the P-MSSS we referred to the previously published Disability Expectancy Table (34).

#### Comorbidities

The Self-Administered Comorbidity Questionnaire (SCQ) is a self-report tool to assess the presence of comorbidities in the absence of medical record review (36). The SCQ has demonstrated criterion validity when assessed against medical records (37), and has been used in studies of participants with MS (38, 39). We modified this tool, retaining the item regarding whether the comorbidity is currently present and omitting items relating to whether treatment is received and if the condition limits activities. For this study, rheumatoid and osteoarthritis conditions were combined into one, and due to anticipated high prevalence, anxiety was also included as a condition.

#### Vision

We assessed participant level of visual impairment using the vision component of the validated Performance Scale (PS-V) (25, 26), used in NARCOMS. Participants were asked to describe their overall visual condition (with glasses if used) over the preceding month, and to compare this current condition to the vision they had before developing MS. The PS-V uses a 6-point scale (“normal vision” to “total visual disability”) with detailed descriptors. It is strongly correlated with the Impact of Visual Impairment Scale, which assesses vision-related quality of life (QoL) by self-report (*r* = 0.66, *p* = 0.0001) (40).

#### Health-Related Quality of Life

The Multiple Sclerosis Quality of Life-54 (MSQOL-54) was used to measure health-related QoL (41). This tool was developed from the RAND 36-item health survey (SF-36) and is supplemented with 18 additional items. The MSQOL-54 consists of 54 items from which 12 sub-scales and two single items are calculated, which together yield two composite scores: the physical health composite (PHC) and mental health composite (MHC).

#### Fatigue

The 9-item Fatigue Severity Scale (FSS) (42) was used to assess clinically significant fatigue. A mean score ≥4 has been used as a cut-off to indicate clinically significant fatigue and is widely used for people with MS (43–45). It has good internal consistency, stability, and sensitivity to change over time (46, 45).

#### Depression

To assess depression risk at 2.5-year follow-up, we used the Patient Health Questionnaire (PHQ-9), a widely used depression risk screening tool validated in people with MS (23, 47). The PHQ-9 has comparable psychometric properties to the CESD-10, and PROMIS-D-8 when tested in people with MS (48). The short version of this tool, the PHQ-2 was used at baseline and comprises two-items of the PHQ-9. We opted for the longer version at follow-up in view of its superior psychometric characteristics. We report those at risk for depression with cut-off at ≥10 on the PHQ-9 (49). For the PHQ-2 (baseline and 2.5-year follow-up) we applied a cut-off of ≥3 (50).

#### Medication Use

A list of 25 disease-modifying drugs (DMDs) and other common MS medications including generic and trade names were provided. Medications for which data were collected were the same as described at baseline with the addition of Peginterferon beta-1a (PLEGRIDY®). Participants were asked to indicate current and previous use, and the length of time taken.

Participants were also asked to indicate whether they took prescription, over-the-counter, or herbal remedies for 10 symptomatic conditions: depression, anxiety, headaches, pain other than headaches, fatigue, difficulty sleeping, bladder problems, bowel problems, spasticity, and “other.”

#### Health Service Use

Participants were asked four questions relating to the frequency of health service use over the preceding 6 months based on items in the Stanford Chronic Disease Self-Management Study (28), the validity of which has been demonstrated (51). Data were collected for visits to a medical doctor, visits to emergency rooms, number of overnight stays in hospital, and total nights spent in hospital.

#### Dietary Habits

To minimize respondent burden, we selected a brief dietary screening tool rather than a food diary or Food Frequency Questionnaire. Since we aimed to assess diet broadly with a particular consideration for fats, we modified the Diet Habits Questionnaire (DHQ) (52). The DHQ was developed in line with nutrition recommendations by the National Heart Foundation of Australia, and the Dietary Guidelines for Australian Adults developed by the National Health & Medical Research Council and the Commonwealth Department of Health and Aging. The original 24-item DHQ has eight dietary sub-scores, and assesses intake of saturated and unsaturated fat, fruit and vegetables, fiber, takeaway foods, snack habits, and omega-3 consumption, among other estimates. For the purpose of the HOLISM study we removed three items regarding sodium intake, and one item on alcohol (53). For questions relating to oils and fats, participants were provided with examples of vegetable oil, mono-unsaturated oil, and polyunsaturated oil to which they could refer. Concurrent validity for the original DHQ has been established for an Australian cardiac disease population (52).

For the 2.5-year timepoint, we added a researcher-devised item regarding oily fish consumption that was separate from the DHQ: “How often do you eat oily fish such as sardines, mackerel, herring, salmon, tuna or trout?,” with available responses including Never; Less than once a week; About 1–2 times a week; About 3–4 times a week; At least 5 times a week.

#### Alcohol Consumption

Frequency of alcohol consumption was assessed on an 11-point ordinal scale (never drink to drink daily), collapsed to a five-point scale (non-drinker, rarely, <1/week, 1–3 days a week, 4 days/week to daily). Data for volume normally consumed were collected on an 11-point scale (ranging from “not applicable” to 10+ standard drinks per day) with examples provided for what constituted a “standard drink.” Based on prior classifications (54), “low” consumption was defined as <15 g/week; “moderate” consumption was defined as 15–210 g/week (or up to 30 g/day) for women and 15–315 g/week (or up to 45 g/day) for men; “high” consumption was considered >210 g/week (30 g/day) for women or >315 g/week (>45 g/day) for men.

#### Smoking

Current smoking status was queried as current/former/never, while number of tobacco products smoked was assessed using six categories (<1 per day, 1–5 per day, 6–10 per day, 11–15 per day, 16–20 per day, to >20 per day). Duration of smoking (years) was collected on an interval scale. Time since quitting if previously smoked was queried as <6-months; 6-<12-months; 1-<2-years; 2-<3-years; 3-<4-years; 4-<5-years; 5-<10-years; more than 10 years ago, subsequently collapsed into three categories (<12-months; 12-months-10-years; 10-years+).

#### Sunlight Exposure

Questions relating to sun exposure were modified from the Ausimmune Longitudinal Study in people with MS (55). For each of summer and winter, participants were asked to report number of days per week they were out in the sun; the average duration spent in the sun on days they were out in the sun (none, 1–15 min, 16–30 min, 31-60 min, >60 min); frequency of sunscreen applied to the majority of exposed skin (never, sometimes, often, always); frequency of wearing clothes that covered most of the body (never, sometimes, often, always); frequency of wearing clothes that exposed much of the body (never, sometimes, often, always).

A researcher-devised item explored participants' 12-month average weekly frequency of “adequate” sun exposure. We defined adequate sun exposure as “10–15 min of sunlight on days with UV index of 7” (more or less time if the UV index was lower or higher). Response options were never, less than once a week, 1–2 times per week, 3–4 times per week, 5–6 times per week, every day, unsure. Also, participants were asked: the color of their untanned skin (very light; light; intermediate; tanned; brown; dark) based on existing classification (56).

#### Vitamin D

Participants were asked whether they intentionally exposed themselves to the sun to raise their vitamin D level; whether they took a vitamin D supplement and, if so, the dosage and frequency of their vitamin D supplementation, and the duration of vitamin D supplementation (<6 months; 6 to <12 months; 1 year to <2 years; 2 to <3 years; 3 to <4 years; 4 to <5 years; 5 to <10 years; ≥10 years).

#### Omega-3 Fatty Acids

Items included both the type and daily dosage of omega-3 supplementation used on average in the last 12 months. Types of omega-3 included fish oil, high-potency fish oil, flaxseed oil, and “other” free-text responses.

#### Physical Activity

We used the International Physical Activity Questionnaire-Short Form (IPAQ-SF), a 7-day recall of the frequency and duration of vigorous and moderate physical activity, walking, and sitting assessed in nine items (57). Items can be scored separately, as a combined total score, or the number of metabolic equivalent of task (MET) minutes can be calculated. Using established scoring instructions, we categorized data into low activity level (no activity reported or insufficient to be other levels), moderate activity level (3+ days of vigorous at least 20 min/day, 5+ days of moderate/walking at least 30 min/day, or any combination of 600+ MET-min/week); and high activity level (at least 1,500 vigorous MET-min/week, at least 3,000 total MET-min/week).The IPAQ and its short form have been validated in several studies and populations globally, and both the long form (58, 59) and the short form (60, 61) have been used previously for people with MS. The short form has also been validated with accelerometers (62).

#### Meditation

Two researcher-devised items measured the 12-month average weekly frequency and duration of meditation: “On average in the last 12 months how often have you meditated” (never; less than once per week; 1–2 times per week; 3–4 times per week; 5–6 times per week; everyday); and “On average, how long do you meditate for (in minutes) each time?.” For the latter, the response format measured duration in 5-min increments up to “more than 60 min.”

#### Social Support

The relationship between social support and health has long been acknowledged (63). We used the Single Item Measure of Social Support (SIMSS) to determine the number of people that provided support to participants (64): “How many people do you have near you that you can readily count on for help in times of difficulty, such as watch over children or pets, give rides to hospital or store, or help when you are sick?” (0, 1, 2–5, 6–9, 10, or more).

#### Engagement With OMS

Three items queried participants' engagement in resources (books, website, and educational workshops) provided by the not-for-profit charitable organization Overcoming Multiple Sclerosis (OMS), for which several of the researchers had previously facilitated lifestyle educational sessions for people with MS. OMS promotes a healthy lifestyle (no smoking, regular vigorous exercise, daily meditation, plant-based whole food diet low in saturated fat, plus seafood and omega-3 supplementation, regular sun exposure and vitamin D supplementation) with the aim of reducing relapses, MS symptoms, and disability accrual. The HOLISM study was conceived by the founder of OMS, and the HOLISM study and OMS are funded by the same charity.

#### Mastery

The Pearlin Mastery Scale (Mastery-S) (24), a 7-item scale, was used to assess the extent to which individuals felt they manifested personal mastery over important life outcomes. Participants were presented with statements regarding their ability to control and master aspects of life and provided four response options: strongly disagree, disagree, agree, or strongly agree. Items were summed to a total score ranging from 7 to 28. The Mastery-S has demonstrated internal reliability both in general populations (0.72) (24) and adults with MS (0.75) (65).

### Data Analysis

For both cohorts, we reported descriptive statistics: number (percentage) for categorical variables; mean (standard deviation) for continuous variables; and median (25–75^th^ percentile) for skewed variables. The baseline characteristics of the participants who completed or dropped-out at 2.5-year follow-up were also reported, and univariable logistic regression was used to explore whether these characteristics are associated with the missingness at the 2.5-year follow-up. All statistical analyses were completed using Stata/SE, version 15.0 (StataCorp, College Park, Texas).

## Results

### HOLISM Longitudinal Cohort, Participation at 2.5-Year Follow-Up

**Figure 1:**
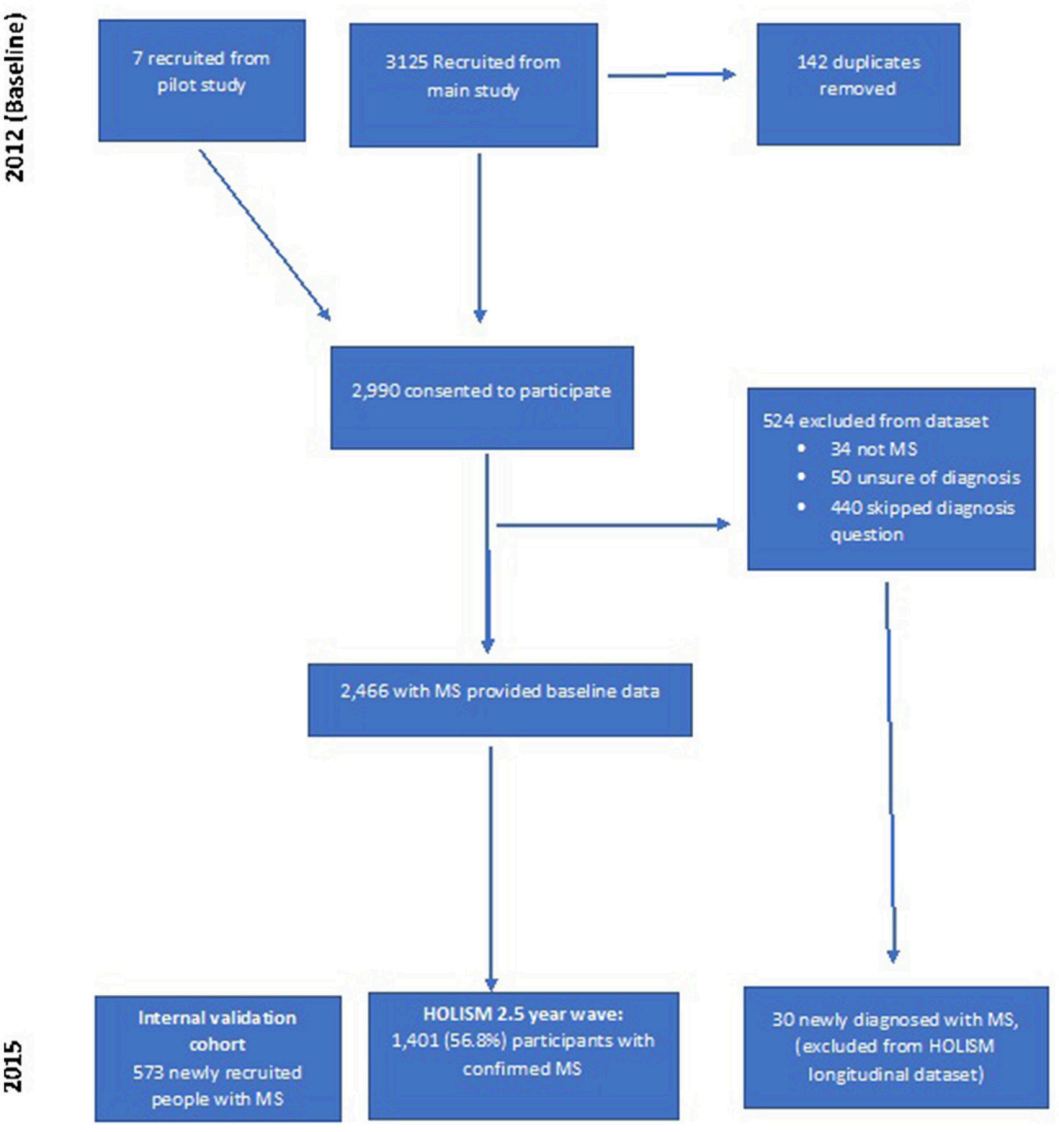
Flowchart of participation.

Of 2,990 that consented to participate at baseline, 524 were excluded leaving 2,466 people with MS at baseline (Figure 1). Of these baseline participants with diagnosed MS, 1,401 (56.8%) provided data at the 2.5-year wave. A further 30 participants that commenced the initial baseline survey but did not report a diagnosis of MS at baseline (and were therefore not included in previous reports of our baseline sample), indicated a subsequent diagnosis of MS at 2.5-year follow-up. Since they did not have definite MS at baseline, these 30 participants were not included in longitudinal analyses.

### HOLISM Longitudinal Cohort, Attrition at 2.5-Year Follow-Up

In total, 1,063 baseline participants did not complete 2.5-year follow-up, 43.2% of the baseline sample with diagnosed MS. Survey completion at 2.5 years was greater for participants who had reported higher educational attainment (Table 1), better health outcomes (Table 2), engagement in OMS resources (Table 3), and healthier lifestyle (Table 4) at baseline.

**Table 1:** Sociodemographic characteristics of the 2.5-year and validation cohorts and the baseline characteristics of the participants who completed and were lost to follow-up at 2.5-year follow-up.

	**Baseline, completed 2.5-year (Number (%) unless stated otherwise) (*n* = 1,401)**	**Baseline, not completed 2.5-year (Number (%) unless stated otherwise) (*n* = 1,065)**	**2.5-year (Number (%) unless stated otherwise) (*n* = 1,401)**	**Validation (Number (%) unless stated otherwise) (*n* = 573)**
**SOCIODEMOGRAPHICS**				
Sex				
Male	241 (17.3)	174 (18.1)	241 (17.3)	102 (19.2)
Female	1,150 (82.7)	787 (81.9)	1,150 (82.7)	430 (80.8)
(Missing)	(10 (0.7))	(104 (9.8) ∧)	(10 (0.7))	(41 (7.2))
**REGION OF RESIDENCE**				
Australasia	560 (40.1)	275 (25.9)	564 (40.3)	199 (34.7)
Europe	380 (27.2)	279 (25.2) ∧	378 (27.0)	234 (40.8)
North America	426 (30.5)	487 (45.8) ∧	430 (30.7)	128 (22.3)
Other	30 (2.2)	33 (3.1) ∧	29 (2.1)	8 (1.40)
(Missing)	(5 (0.4))	(2 (0.2))	(0 (0))	(4 (0.70))
**MARITAL STATUS**				
Married	856 (61.8)	615 (59.0)	857 (62.3)	345 (60.2)
Cohabiting/partnered	195 (14.1)	121 (11.6)	190 (13.8)	88 (15.4)
Separated/divorced/widowed	147 (10.6)	136 (13.1)	168 (12.2)	60 (10.5)
Single	187 (13.5)	170 (16.3)	160 (11.6)	75 (13.1)
(Missing)	(16 (1.1))	(23 (2.2))	(26 (1.9))	(5 (0.9))
**NUMBER OF CHILDREN**				
None	473 (33.8)	342 (32.1)	442 (31.6)	222 (38.4)
One or more	904 (64.5)	694 (65.2)	923 (65.9)	341 (59.5)
(Missing)	(24 (1.7))	(29 (2.7))	(36 (2.6))	(10 (1.8))
**NUMBER OF SUPPORT PEOPLE**				
None	51 (3.6)	66 (6.2)	43 (3.1)	23 (4.0)
One	267 (19.1)	210 (19.7)	271 (19.3)	101 (17.6)
Two to five	810 (57.8)	518 (48.6)	795 (56.8)	316 (55.2)
Six or more	194 (13.9)	107 (10.1)	156 (11.1)	63 (11.0)
(Missing)	(79 (5.6))	(164 (15.4) ∧)	(136 (9.7))	(70 (12.2))
**EMPLOYMENT**				
Unemployed, not seeking employment	61 (4.4)	63 (6.0)	60 (4.4)	29 (5.1)
Retired due to age	55 (3.9)	18 (1.7)	79 (5.7)	19 (3.3)
Retired for medical/disability reasons	277 (19.8)	297 (28.1) ∧	336 (24.4)	107 (18.7)
Other	13 (0.9)	9 (0.9)	17 (1.2)	7 (1.2)
(Missing)	(3 (0.2))	(7 (0.7))	(23 (1.6))	(1 (0.2))
**LEVEL OF EDUCATION COMPLETED**				
No formal schooling/primary/secondary only	292 (21.0)	311 (29.3)	268 (19.4)	135 (23.6)
Vocational school	203 (14.6)	193 (18.2)	212 (15.4)	90 (15.7)
Bachelor's degree	533 (38.3)	354 (33.4)	516 (37.4)	207 (36.1)
Post-graduate study	364 (26.2)	202 (19.1	385 (27.9)	139 (24.3)
(Missing)	(9 (0.6))	(5 (0.5))	(20 (1.4))	(2 (0.4))
Age^*^	45.9 (10.5)	45.3 (10.5)	48.4(10.5)	46.2 (11.0)
(Missing, *n* (%))	(0 (0.0))	(38 (3.6))	(0 (0.0))	(0 (0.0))
Latitude^*^	41.0 (8.8)	41.0(8.6)	41.0 (8.8)	42.6 (8.9)
(Missing, *n* (%))	(3 (0.2))	(2 (0.2))	(0 (0.0))	(0 (0.0))
Comparative socioeconomic status^**^	-	-	5 (5-6)	6 (5–6)

* *Mean (standard deviation)*.

** *Median (25–75th percentile). The baseline characteristics that are associated with missingness at 2.5 year follow-up are highlighted in gray with ∧ indicating the variables that are associated with higher odds of missingness at 2.5 year follow-up. For categorical variables, the reference category was taken as the first presented category in the table, and an arbitrary p-value cut-off of 0.05 was used*.

**Table 2:** Anthropometric and clinical characteristics of the 2.5-year and validation cohorts and the baseline characteristics of the participants who completed and were lost to follow-up at 2.5-year follow-up.

	**Baseline, completed 2.5-year (Number (%) unless stated otherwise) (*n* = 1,401)**	**Baseline, not completed 2.5-year (Number (%) unless stated otherwise) (*n* = 1,065)**	**2.5-year (Number (%) unless stated otherwise) (*n* = 1,401)**	**Validation (Number (%) unless stated otherwise) (*n* = 573)**
**ANTHROPOMETRIC AND CLINICAL**				
Body Mass Index				
Underweight (<18.5)	71 (5.1)	35 (3.4)	60 (4.3)	31 (5.4)
Normal (18.5–<25)	792 (56.6)	501 (48.1)	781 (55.8)	299 (52.2)
Overweight (25 – <30)	304 (21.7)	252 (24.2)	304 (21.7)	126 (22.0)
Obese (≥30)	232 (16.6)	253 (24.3) ∧	254 (18.2)	107 (18.7)
(Missing)	(2 (0.1))	(24 (2.3) ∧ )	(2 (0.1))	(10 (1.75))
**NUMBER OF COMORBIDITIES**				
0	516 (36.8)	340 (31.9)	715 (51.0)	321 (56.0)
1	392 (28.0)	232 (21.8)	366 (26.1)	131 (22.9)
2	270 (19.3)	216 (20.3)	182 (13.0)	61 (10.7)
≥3	223 (15.9)	277 (26.0) ∧	138 (9.9)	60 (10.5)
**CURRENTLY USE A DISEASE MODIFYING DRUG?**				
No	747 (53.3)	574 (53.9)	812 (58.0)	310 (54.1)
Yes	654 (46.7)	491 (46.10)	589 (42.0)	263 (45.9)
**DISABILITY LEVEL (PATIENT-DETERMINED DISEASE STEPS)**				
Normal/mild	787 (56.2)	476 (44.7)	783 (55.9)	343 (59.9)
Moderate	442 (31.6)	353 (33.2) ∧	400 (28.6)	169 (29.5)
Severe	117 (8.4)	123 (11.6) ∧	177 (12.6)	48 (8.4)
(Missing)	(55 (3.93))	(113 (10.6) ∧)	(41 (2.9))	(13 (2.3))
Mental Health Composite Quality Of Life (MSQOL-54) ^*^	70.3 (19.6)	61.6 (22.7)	70.3 (20.4)	68.9 (20.6)
(Missing, *n* (%))	(74 (5.3))	(122 (11.5) ∧)	85 (6.07)	(46 (8.0))
Physical Health Composite Quality Of Life (MSQOL-54) ^*^	63.4 (32.3)	54.6 (33.8)	60.9 (30.4)	65.1 (32.8)
(Missing, *n* (%))	(42 (3.0))	(68 (6.4) ∧)	43 (3.07)	(12 (2.1))
**DEPRESSION RISK (PATIENT HEALTH QUESTIONNAIRE−9 SCORE)**				
0 – 4: no depression	-	-	607 (48.0)	245 (42.8)
5–9: minimal depression symptoms			383 (30.3)	134 (23.4)
10–14: likely major depression, mild			144 (11.4)	81 (14.1)
15–19: likely major depression, moderate			93 (7.4)	26 (4.5)
≥20: likely major depression, severe			37 (2.9)	21 (3.7)
(Missing)			(137 (9.8))	(66 (11.5))
**DEPRESSION RISK (PATIENT HEALTH QUESTIONNAIRE−2 SCORE)**				
0–2: negative	1139 (81.3)	660 (62.0)	1119 (79.9)	441 (77.0)
≥3: positive	181 (12.9)	244 (23.0) ∧	190 (13.6)	82 (14.3)
(Missing)	(81(5.8))	(161 (15.1) ∧)	(92(6.6))	(50 (8.7))
**FATIGUE, AS DEFINED BY FATIGUE SEVERITY SCALE** **>35**				
No fatigue	484 (34.6)	251 (23.6)	476 (37.5)	201 (35.1)
Fatigue	784 (56.0)	619 (58.1)	792 (62.5)	308 (53.8)
(Missing)	(133 (9.5))	(195 (18.3))	(133 (9.5))	(64 (11.2))
Pearlin Mastery Scale^**^	-	-	21 (19-25)	21 (18-24)
(Missing)			(146 (10.4))	(71 (12.4))
**PERFORMANCE SCALE—VISION COMPONENT (PS-V)**				
Normal			603 (43.0)	225 (39.3)
Minimal			430 (30.7)	156 (27.2)
Mild			201 (14.4)	95 (16.6)
Moderate			57 (4.1)	23 (4.0)
Severe			11 (0.8)	10 (1.8)
Total			1 (0.1)	1 (0.2)
(Missing)			(98 (7.0))	(63 (11.0))

* *Mean (standard deviation)*.

** *Median (25–75th percentile)*.

*The baseline characteristics that are associated with missingness at 2.5-year follow-up are highlighted in gray with ∧ indicating the variables that are associated with higher odds of missingness at 2.5-year follow-up. For categorical variables, the reference category was taken as the first presented category in the table, and an arbitrary p-value cut-off of 0.05 was used*.

**Table 3:** MS-Specific characteristics of the 2.5-year and validation cohorts and the baseline characteristics of the participants who completed and were lost to follow-up at 2.5-year follow-up.

	**Baseline, completed 2.5-year (Number (%) unless stated otherwise) (*n* = 1,401)**	**Baseline, not completed 2.5-year (Number (%) unless stated otherwise) (*n* = 1,065)**	**2.5-year (Number (%) unless stated otherwise) (*n* = 1,401)**	**Validation (Number (%) unless stated otherwise) (*n* = 573)**
**MS-SPECIFIC**				
Type Of Ms At Completion Of Survey				
Benign	64 (4.6)	36 (3.5)	85 (6.2)	19 (3.3)
RRMS	875 (63.3)	616 (59.4)	810 (59.2)	386 (67.4)
SPMS	144 (10.4)	131 (12.6)	199 (14.6)	59 (10.3)
PPMS	100 (7.2)	75 (7.2)	111 (8.1)	40 (7.0)
PRMS	18 (1.3)	30 (2.9)	23 (1.7)	5 (0.9)
Unsure/other	181 (13.1)	149 (14.4)	140 (10.2)	53 (9.3)
(Missing)	(19 (1.4))	(28 (2.6))	(33 (2.4))	(11 (1.9))
Patient-derived Multiple Sclerosis Severity Score^**^	4.4 (2.4–7.3)	5.5 (2.9-7.6) ∧	4.9 (2.6 – 7.3)	4.5 (3.0-7.7)
(Missing, *n* (%))	(66 (4.7))	(132 (12.4) ∧ )	(49 (3.5))	(25 (4.4))
Disease duration since diagnosis, years^**^	5.4 (2.4–11.4)	6.4 (2.6–12.4) ∧	7.9 (5.0–13.9)	4.8 (2.1–12.2)
(Missing, *n* (%))	(0 (0.0))	(29 (2.7) ∧ )	(0 (0.0))	(8 (1.4))
Disease duration since symptom onset, years^**^	11.4 (5.4–20.2)	12.4 (6.6−20.6) ∧	14.2 (8.1–23.2)	11.2 (5.2,20.2)
(Missing, *n* (%))	(2 (0.1))	(37 (3.5) ∧ )	(3 (0.2))	(10 (1.7))
Number of doctor-diagnosed relapses in previous 12 months in relapsing-remitting MS ^**^	0 (0–1)	0 (0–1)	0 (0–1)	0 (0-1)
(Missing, n (%))	(28 (3.2))	(17 (2.8))	(18 (2.2))	(2 (0.5))
**CURRENTLY EXPERIENCING SYMPTOMS DUE TO A RECENT RELAPSE IN RELAPSING-REMITTING MS**				
No	535 (61.1)	360 (58.4)	576 (71.1)	229 (59.3)
Yes	225 (25.7)	190 (30.8)	147 (18.2)	112 (29.0)
Unsure	114 (13.0)	64 (10.4)	80 (9.9)	43 (11.1)
(Missing)	(1 (0.1))	(2 (0.3))	(7 (0.9))	(2 (0.5))
**OVERCOMING MULTIPLE SCLEROSIS (OMS) ENGAGEMENT**				
No OMS resources	350 (25.0)	445 (41.8)	335 (23.9)	149 (26.0)
Website only	257 (18.3)	239 (22.4)	193 (13.8)	101 (17.6)
Book only	43 (3.1)	30 (2.8)	55 (3.93)	10 (1.8)
Retreat only	1 (0.1)	1 (0.1)	2 (0.1)	2 (0.4)
Book and website only	576 (41.1)	278 (26.1)	591 (42.2)	219 (38.2)
Website and retreat only	2 (0.1)	3 (0.3)	1 (0.1)	4 (0.7)
Book and retreat only	5 (0.4)	8 (0.8)	7 (0.5)	0 (0)
Book, retreat and website	167 (11.9)	61 (5.7)	217 (15.5)	88 (15.4)
**HEALTH SERVICE UTILIZATION, PAST 6 MONTHS**				
Number of visits to a medical doctor^**^			2 (1-3)	2 (1-4)
**VISITS TO EMERGENCY DEPARTMENT**				
No			1,140 (81.4)	424 (74)
Yes			154 (11)	102 (17.8)
(Missing)			(107 (7.6))	(47 (8.2))
**OVERNIGHT STAYS IN HOSPITAL**				
Yes			87 (6.2)	52 (9.1)
Number of nights			0(0-0)	0 (0-0)
No			1,196 (85.344)	468 (81.7)
(Missing)			(118 (8.4))	(53 (9.3))
**MS IMPACTED MY EMPLOYMENT**				
No			516 (36.8)	230 (40.1)
Yes			842 (60.1)	330 (57.6)
(Missing)			(43 (3.1))	(13 (2.3))

^*^Mean (standard deviation).

** *Median (25-75th percentile)*.

*^#^ = The baseline characteristics that are associated with missingness at 2.5 year follow-up are highlighted in gray with ∧ indicating the variables that are associated with higher odds of missingness at 2.5 year follow-up. For categorical variables, the reference category was taken as the first presented category in the table, and an arbitrary p-value cut-off of 0.05 was used*.

**Table 4:** Lifestyle characteristics of the 2.5-year and validation cohorts and the baseline characteristics of the participants who completed and were lost to follow-up at 2.5-year follow-up.

	**Baseline, completed 2.5-year (Number (%) unless stated otherwise) (*n* = 1,401)**	**Baseline, not completed 2.5-year (Number (%) unless stated otherwise) (*n* = 1,065)**	**2.5-year (Number (%) unless stated otherwise) (*n* = 1,401)**	**Validation (Number (%) unless stated otherwise) (*n* = 573)**
**LIFESTYLE**				
Smoking Status				
Never	707 (50.5)	392 (36.8)	701 (50.0)	275 (48.0)
Ex-smoker	520 (37.1)	388 (36.4)	527 (37.6)	216 (37.7)
Current smoker	114 (8.1)	167 (15.7)	102 (7.3)	43 (7.5)
(Missing)	(60 (4.3))	(118 (11.1))	(71 (5.1))	((39 (6.8))
**ALCOHOL INTAKE**				
No alcohol intake	516 (36.8)	478 (44.9)	461 (32.9)	184 (32.1)
Limited (1 std/d female, 2 std/d male)	631 (45.0)	339 (31.8)	637 (45.5)	256 (44.7)
Heavy (>1 std/d female, >2 std/d male)	180 (12.9)	106 (9.9)	157 (11.2)	78 (13.6)
(Missing)	(74 (5.3))	(142 (13.3) ∧)	(146 (10.4))	(55 (9.6))
**PHYSICAL ACTIVITY (INTERNATIONAL PHYSICAL ACTIVITY QUESTIONNAIRE)**				
Low	419 (29.9)	328 (30.8)	396 (28.3)	126 (22.0)
Moderate	540 (38.5)	310 (29.1)	563 (40.2)	222 (38.7)
High	316 (22.6)	217 (20.4)	314 (22.4)	156 (27.2)
(Missing)	(126 (9.0))	(210 (19.7) ∧)	(128 (9.1))	(69 (12.0))
**ADEQUATE SUN EXPOSURE IN PRECEDING 12 MONTHS**				
Never/<once per week	392 (31.8)	263 (31.1)	298 (25.4)	124 (22.3)
1–2 times per week	368 (29.9)	223 (26.4)	320 (27.2)	115 (22.1)
3–4 times per week	258 (20.9)	182 (21.5)	306 (26.0)	126 (22.0)
5–6 times per week	133 (10.8)	86 (10.2)	169 (14.4)	65 (11.3)
Every day	81 (6.6)	92 (10.9)	82 (7.0)	39 (6.8)
(Missing)	(169 (12.1))	(219 (20.6))	(226 (16.1))	(100 (17.4))
Diet Habits Questionnaire total score^**^	82 (73–91)	77 (67–85)	80 (70–89)	84.5 (72 92)
(Missing)	(55 (3.9))	(118 (11.8)∧)	(69 (4.9))	(37 (6.4))
**VITAMIN D**				
None	279 (19.9)	343 (32.2)	278 (19.8)	112 (19.6)
<2000 International Unit (IU)/day	188 (13.4)	114 (10.7)	144 (10.3)	49 (8.6)
2000-5000IU/day	258 (18.4)	204 (19.2)	233 (16.6)	84 (14.7)
5000+ IU/day	658 (47.0)	376 (35.3)	727 (51.9)	313 (54.6)
(Missing)	(18 (1.3))	(28 (2.6) ∧)	(19 (1.4))	(15 (2.6))
**OMEGA 3 SUPPLEMENTATION**				
None	469 (33.5)	529 (49.7)	546 (39.0)	209 (36.5)
Fish oil only	469 (33.5)	312 (29.3)	355 (25.3)	94 (16.4)
Flaxseed only	145 (10.4)	55 (5.2)	274 (19.6)	167 (29.1)
Both marine and plant-based omega-3	278 (19.8)	139 (13.1)	187 (13.4)	85 (14.8)
Other	40 (2.9)	30 (2.8)	39 (2.8)	18 (3.1)
**MEDITATION**				
Never	592 (42.3)	477 (44.8)	532 (38)	175 (30.5)
<1 times/wk.	301 (21.5)	196 (18.4)	318 (22.7)	133 (23.2)
1–4 times/wk.	288 (20.6)	159 (14.9)	288 (20.6)	134 (23.4)
>5 times/wk.	148 (10.6)	82 (7.7)	169 (12.1)	73 (12.7)
(Missing)	(72 (5.1))	(151 (14.2)∧)	(94 (6.7))	(58 (10.1))

^*^Mean (standard deviation).

** *Median (25th-75th percentile)*.

*^#^The baseline characteristics that are associated with missingness at 2.5 year follow-up are highlighted in gray with ∧ indicating the variables that are associated with higher odds of missingness at 2.5 year follow-up. For categorical variables, the reference category was taken as the first presented category in the table, and an arbitrary p-value cut-off of 0.05 was used*.

### HOLISM Longitudinal Cohort, Characteristics at 2.5-Year Follow-Up

Participants in the HOLISM longitudinal cohort (completing baseline and 2.5-year follow-up) were predominantly female, residing in Australasia, Europe or North America, married, and either employed or retired due to medical reasons or disability (Table 1). Participants were highly educated, with approximately two thirds having completed a tertiary level study. Most had relapsing-remitting MS (RRMS) (Table 3), and 29% reported use of a cane or disability affecting gait (Table 2). Those retained in the study fluctuated in disability across the 2.5 year follow-up period [normal/mild −4 (−0.3%); moderate −42 (−3.0%); severe +60 (+4.2%); missing −14 (−1.03%)].

Approximately 40% were overweight or obese, and most were physically active to a moderate or high level. Most participants were ex-smokers or never smokers, consumed low amounts of alcohol, and used vitamin D and/or omega-3 fatty acid supplementation. At the time of survey completion, 42% reported currently taking a disease-modifying drug (Table 4).

Half of the respondents of the 2.5-year wave reported having a comorbidity, and the rate of clinically significant fatigue was high (62.5%). One-fifth screened positive for depression (PHQ-9 at cut off ≥10), and 50% reported some level of visual impairment (Table 2).

### Validation Cohort Participation and Characteristics

We recruited 573 participants into the HOLISM validation cohort. Overall, this cohort was similar to the original HOLISM baseline cohort (14) but small differences were seen in number of children (Table 1), MS type (Table 3), vitamin D supplementation, and use of meditation for stress reduction (Table 4).

## Discussion

The importance of lifestyle has only relatively recently been identified by key stakeholders as a critical area for further research (66, 67). A comprehensive body of literature exists that suggests modifiable risk factors, such as lifestyle, play a pivotal role in MS disease progression and associated morbidity (12, 4, 68–72). Yet there remain critical gaps in the literature with respect to modification of these factors. Multiple large national and international MS registries exist that collect longitudinal data (73), several of which have also begun to collect data regarding lifestyle and/or environment. However, our HOLISM study is the first to examine a broad range of lifestyle factors together with disease characteristics, medication use, and health outcomes in a worldwide sample.

Longitudinal research designs are arguably of great importance in the initial identification of exposure-outcome associations within populations. The longitudinal design offers advantages, including the capacity to separate change over time within participants (i.e., effects of time or age) from differences between participants at baseline (i.e., cohort effects), as well as the determination of temporal relationships permitting inference regarding prospective interpretations (74). Findings from epidemiological studies such as ours may instigate longitudinal RCTs, the gold standard in establishing causation. We previously reported baseline findings from the HOLISM study which revealed a broad exposure gradient in terms of lifestyle behavior adopted by this international sample, rendering the sample a powerful tool to identify such associations. The current paper reports on follow-up data for the HOLISM sample collected at the 2.5-year wave, and data from a validation sample.

### HOLISM Longitudinal Cohort

#### Participation and Attrition

Despite our attempts to minimize survey dropout by sending multiple email reminders to participants and advertising the follow-up wave through websites and social media used to recruit the baseline survey, our attrition rate was high. Of the 2,466 with confirmed MS that provided baseline data, 1,401 completed the survey at 2.5-year follow-up, an overall response rate of 56.8% for those included in baseline analyses. While there are many possible reasons for attrition, it is likely that the web-based nature of recruitment, the geographically diverse nature of the cohort and limited contact information all contributed to attrition; a single email address was collected from participants at baseline. Participant attrition, an inherent characteristic of almost every longitudinal study, represents a considerable threat to validity; retaining a large, representative portion of the original sample is critical both for statistical power and generalizability (75) and to minimize the potential for selection bias in analyses of associations between exposures and outcomes if participants who are retained in the study are systematically different to those who are lost to follow up. We explored the baseline characteristics of those who participated in the 2.5-year follow-up compared to those who did not. In general, our retained sample was healthier, more educated, and more often employed than those not retained. This notable participation bias is a commonly encountered limitation in most longitudinal studies, which can, in some instances, be managed or minimized using modern missing data methods in multivariable models (76). The impact of these biases on results will be addressed in consequent papers using these data.

Specifically, compared to those missing at 2.5-years, those that completed 2.5-year follow-up reported substantially greater baseline frequencies of residing in Australasia, screening negative for depression risk, and higher mental and physical health quality of life. Among completers, there were substantially higher baseline frequencies of taking an omega-3 and/or vitamin D supplement, and being engaged in OMS resources. Compared with completers, those lost to follow-up were more likely to have indicated that they were more disabled, retired due to medical reasons or disability, obese, have less healthy dietary habits, higher consumption of alcohol, three or more comorbidities, higher MS severity, and longer disease duration. Together these data suggest that non-completion in our HOLISM longitudinal cohort may be associated with poorer health status, poorer lifestyle, greater disease severity and longer duration of the disease.

While we used strategies to minimize survey dropout, it is notable that a greater number of participants in Australasia indicated engagement with the OMS website at baseline; this may have increased bias toward healthy lifestyle in this second wave. This suggests that people who participated in the follow up study are unlikely to be representative of the baseline HOLISM survey.

#### Characteristics of 2.5-Year Sample

At the 2.5-year wave of the HOLISM longitudinal study we retained a geographically diverse range of English speaking participants based predominantly in Australasia, Europe, or North America. We retained a high proportion of females at the 2.5-year follow-up (4.8:1 female:male ratio), which is an over-representation from the estimated incidence sex ratio for people living with MS (female:male) of 2.3–3.5:1 (77), and differs from samples thought to be representative (78). This is unsurprising since previous studies have demonstrated higher participation rates by women in web-based studies (79) and survey research generally. Our sample reported a median of 7.9 years duration since diagnosis. While secondary progressive MS takes many years to develop (80), our cohort is approaching a time at which some may begin to experience changes in MS specific symptoms, comorbidities, and disability (81), offering us the opportunity to explore associations between these health outcomes and exposures.

Our cohort is highly educated, with the majority reporting having completed a bachelor or higher degree. This is relevant since there is known to be a strong association for education with health behavior, health status, and health literacy including the application of information about prevention and treatment (82). Among people with MS, higher education levels are associated with better health care engagement (83), cognitive reserve (84), superior health related QoL (85), and lower levels of disability (86), although these latter two findings were not confirmed in one large registry study (87). A substantial proportion of our population were retired due to disability or illness, and most participants indicated that MS had impacted on their employment. These are important surrogates for the significant burden of disease of MS.

Consistent with what may be expected from prevalence statistics, the majority (*n* = 810, 59%) of our participants reported having RRMS. Notably, only 42% (589) of participants in our sample reported current DMD use. Possible underlying reasons for this require further exploration. It is conceivable that our observation is influenced by the fact that many participants came from countries where DMDs are not reimbursed by healthcare systems. Regardless, these findings are concerning given that delayed initiation of DMD treatment is associated with a more rapid disease progression and mortality (88). Interestingly, 140 participants were unsure of their MS type, despite, re-confirming MS diagnosis on study entry; this number is not dissimilar to that reporting a relapse at the time of the survey completion (*n* = 147). It is possible that at least a subset of participants unsure of their disease status were awaiting either symptom remission or neurology review to determine classification. However, we cannot exclude the possibility that some did not have MS.

Lifestyle and health behavior of the cohort were typically favorable. In contrast to other studies (89), very few smokers participated. Most participants consumed low to moderate amounts of alcohol, were using vitamin D and/or omega-3 supplements, reported healthy diet, and most reported being physically active to a moderate or high level.

Despite the positive health behavior reported by our participants, nearly 40% were overweight or obese. While this is lower than the numbers reported for the general population (90) and for others with MS (91), these data are concerning given the putative role of adiposity as a prognostic indicator in MS (92).

In terms of health outcomes, our cohort reported high rates of morbidity and comorbidity, a pattern that is well-documented for people with MS (93). Approximately half of the respondents reported having at least one comorbidity on the SCQ, and the high rate of clinically significant fatigue was in the range expected for this population (94, 95). One fifth screened positive for depression (PHQ-9); these are similar to findings of the large NARCOMS cohort (96), and comparable to systematic review findings (97). Five percent of our cohort reported moderate to total visual impairment which is slightly lower than that reported by NARCOMS participants with a similar time since diagnosis (81, 98). Collectively, these findings have important implications since physical and psychiatric comorbidities are important determinants of disability progression (99, 100). Compared to the NARCOMS study, our sample reported comparable levels of disability given their time since diagnosis (81, 96). At a median 7.9 years since diagnosis, 12.6% of our sample reported severe disability (bilateral support, wheelchair, or bedridden) compared to 12% of the NARCOMS sample at 8 years post diagnosis (96). For our sample this represented an increase of 60 participants transitioning to the severe disability range over the 2.5 year follow-up.

### Validation Sample

Validation samples are useful tools to assess reproducibility of findings and performance of prognostic models. Validation processes have been extensively described (21, 22, 101, 102). Our methodology, wherein participants were recruited via the same methods as the HOLISM longitudinal study but lagged temporally, provides us with the opportunity to investigate the reproducibility of our findings from the original cohort, and to use in validation of prediction models derived from the HOLISM cohort.

A total of 573 new participants (reporting confirmed MS) provided data in 2015 forming our validation sample. With some exceptions, our data suggest that participants in the validation sample were similar to those that comprise the HOLISM baseline sample in terms of health outcomes and exposures. In most cases differences observed were small, suggesting a reasonable level of internal consistency with the HOLISM longitudinal sample at baseline, which employed similar recruitment and sampling methodology.

Compared to all main sample HOLISM baseline participants described previously (14), the validation sample was comparable in terms of age, sex and, level of education. Our validation sample comprised a greater proportion of people residing in Europe and Australasia and a lower proportion in North America. Compared with HOLISM baseline data (14), the validation sample comprised fewer participants with one or more children (60 vs. 76%), slightly more engaged in full or part-time employment (60 vs. 54%). Validation participants were more likely to report having relapsing remitting MS compared to the HOLISM main baseline sample (67 vs. 62%), but were comparable in time since diagnosis.

For health outcomes, those in the validation sample were more likely to rate their level of disability as normal/mild (60%) compared to in the main HOLISM sample at baseline (54% (14). The samples were comparable in terms of health-related QoL, but not clinically significant fatigue and depression (PHQ-2) for which the validation cohort was less affected (fatigue 54 vs. 66%; depression risk, positive: 14 vs. 19%).

In terms of lifestyle, validation sample participants reported data that were highly comparable to the HOLISM baseline in dietary habits (53), social support, sun exposure, rate of smoking, omega-3 and vitamin D supplementation (14).

In summary, our validation sample, employed similar recruitment and sampling methodology, showed small differences compared to the HOLISM longitudinal sample at baseline. With its temporal separation from the HOLISM cohort, our validation sample renders it suitable for use in temporal or “narrow” validation methods of observations and models derived from the HOLISM cohort. External validation using samples recruited elsewhere and by different researchers would further strengthen our confidence in the transportability of HOLISM findings and any models derived from these data.

### Strengths and Limitations

The HOLISM longitudinal study and validation cohort are not without limitations. The data were self-reported and no confirmation was possible using medical records given the geographical locations of participants around the world. Hence, it is possible that some participants self-reporting doctor-diagnosed MS did not have MS. While self-reported diagnosis has been shown to be valid in other MS cohorts (103), confirmation of diagnoses through medical record checks for a subset of participants from the HOLISM study would be valuable. Measurement error, potentially leading to information bias may therefore have impacted on the accuracy of some findings. Our data may also have been biased by “panel conditioning,” wherein respondents unconsciously change their responses to match the anticipated goals of the research. The online recruitment methodology may have biased our sample; previous research has demonstrated that participants of online research are of higher socioeconomic status. Our sample only includes English-speaking participants, and those able to use a device on which to complete the online survey. People with MS who have significantly impaired vision, dexterity or cognitive ability were thus less likely to have participated. Furthermore, the online recruitment focussed mostly on websites, Facebook pages and forums with a healthy lifestyle focus. Finally, the length of our survey, at approximately 40 min, may have been arduous for some participants experiencing fatigue or significant disability. We attempted to minimize this by enabling participants to save their responses and re-enter the survey on separate occasions. Participation bias is therefore a factor, further enhanced by those with healthier lifestyles being more likely to both participate and complete follow-up. However, our data on a range of lifestyle, sociodemographic and clinical covariates will enable us to control for these biases to some extent but the results are unlikely to be representative of all people diagnosed with MS.

For the HOLISM longitudinal cohort, we observed appreciable attrition over the 2.5-year study duration (104). In contrast to other studies we did not use incentives to maximize retention. It is not possible to determine the degree to which attrition was due to unwillingness to participate, changes in contact information, or death given no information is available on those lost to follow-up. Attrition was not random and loss to follow-up may have further contributed to non-representativeness in our cohort, which was already not a random, or representative sample of people with MS at baseline.

Together, these limitations may result in biased estimates and some associations may be less representative of the global MS population. Due to high attrition and associated non-representativeness, comparisons between subgroups within our sample may be limited. While attrition has resulted in a sample that is healthier and more highly educated, and may reduce representativeness further, healthy volunteer bias is a commonly documented limitation in longitudinal research. This is likely to be further complicated by the lack of diversity of participants recruited into cohort studies such as this wherein the heterogeneity of people with MS is not fully represented. This is seen in other MS cohorts such as NARCOMS which may under-represent those with mild and severe impairments (96). Interestingly, while selective recruitment into cohort studies can impact baseline prevalence characteristics of the recruited sample compared to the wider eligible population (105–107), simulation studies (108) and empirical studies (109) indicate that such baseline selectivity does not necessarily impact on the validity of associations between baseline exposures and longitudinal outcomes.

The prevalence of relapse-related symptoms at survey completion was similar for the main sample at baseline and the validation cohort (27 and 29%, respectively) (14). It is conceivable that those experiencing a relapse may have reported data that were not indicative of their usual health status, instead reporting higher morbidity in health outcomes than those not experiencing a relapse. Accordingly, we can consider this covariate in the analysis to gauge what impact relapse-associated symptomatology has, and potentially to undertake sensitivity analyses restricted to those without such ongoing relapse symptoms.

The HOLISM and validation cohorts have several strengths that deserve acknowledgment. They are strengthened by the wide exposure gradient apparent for lifestyle and demographics, which will better enable associations to be identified. Further comparison to other longitudinal datasets may provide further confirmation of exposure gradients. The longitudinal nature of the data may reveal “sleeper effects,” that is, those that are detectable only over a long period of time, and which may not be detectable in cross-sectional studies (110). The number of variables collected, together with our large sample size enable us to adjust for many potential confounders. We recruited and retained participants from a broad range of countries of birth and locations of residence. Finally, wherever possible we used validated tools to minimize measurement bias, many of which had been used previously for people with MS.

## Conclusions

Our HOLISM longitudinal data have substantial utility in enabling the exploration of associations between a large range of variables and, importantly, their change over time. Strategies to account for bias due to high and selective attrition will be an important consideration in future papers reporting on analyses from this dataset. Our validation sample will allow us to replicate exposure-outcome associations observed for our longitudinal sample, and to undertake temporal validation of models derived from HOLISM. Ultimately, identifying associations between changes in health behavior and health outcomes over time within participants, may lead to a better understanding of which modifiable factors are associated with better health outcomes in people with MS.

## Author Contributions

TW, CM, GJ, KT, and SN are responsible for study concept, and TW, AD, CM, GJ, ZA, and SS were responsible for decisions about the included content of the paper. AD undertook data analyses and contributed extensively to drafting the manuscript; SS, CM, and GJ provided extensive comments on early versions of the manuscript; KT, SN, WB, EO, ZA, CM, and GJ edited earlier versions of the manuscript; all authors approved the final version of the manuscript.

### Conflict of Interest Statement

GJ, KT, and SN have all received remuneration for facilitating lifestyle courses for people with MS. GJ receives royalties for the books, “Overcoming Multiple Sclerosis: The evidence based 7 step recovery program”; “Overcoming Multiple Sclerosis: The evidence based guide to recovery”; and “Taking Control of Multiple Sclerosis.” The remaining authors declare that the research was conducted in the absence of any commercial or financial relationships that could be construed as a potential conflict of interest.
